# Low Expression of CD5 and CD6 Is Associated with Poor Overall Survival for Patients with T-Cell Malignancies

**DOI:** 10.1155/2022/2787426

**Published:** 2022-08-09

**Authors:** Songnan Sui, Zhiyan Li, Jiaxiong Tan, Liang Wang, Gengxin Luo, Chengwu Zeng, Oscar Junhong Luo, Cunte Chen, Yangqiu Li

**Affiliations:** ^1^Key Laboratory for Regenerative Medicine of Ministry of Education, Institute of Hematology, School of Medicine, Jinan University, Guangzhou 510632, China; ^2^Department of Hematology, First Affiliated Hospital, Jinan University, Guangzhou 510632, China; ^3^Department of Oncology, First Affiliated Hospital, Jinan University, Guangzhou 510632, China; ^4^Department of Systems Biomedical Sciences, School of Medicine, Jinan University, Guangzhou 510632, China

## Abstract

**Background:**

T-cell malignancies (TCMs), including T-cell acute lymphoblastic leukemia (T-ALL) and T-cell lymphoma (TCL), are highly aggressive and have a poor prognosis. To further understand prognostic stratifications and to design targeted therapies, this study aims to explore novel, potential biomarkers based on alterations in immune costimulatory molecules (CMs) for TCMs.

**Methods:**

Peripheral blood from 25 *de novo* T-ALL patients in our clinical center and transcriptome data from 131 to 162 patients with peripheral TCL (PTCL) from the GSE19069 and GSE58445 dataset, respectively, were obtained to assess the expression levels of CMs and their prognostic significance.

**Results:**

Seven CMs were associated with overall survival (OS). Among these CMs, CD5 and CD6 had the highest pairwise positive correlation (*R* = 0.69). CD5 and CD6 were significantly down-regulated in TCM patients compared with healthy individuals (HIs), and lower CD5 and CD6 expression was associated with poor OS for both T-ALL and TCL patients, particularly for patients greater than 60 years old. Furthermore, CD5 was positively correlated with CD6 in TCM patients. Compared with patients who were CD5^high^CD6^high^, T-ALL and TCL patients who were CD5^low^CD6^low^ had poor OS. Importantly, CD5^high^CD6^high^ was an independent prognostic predictor for OS in T-ALL (HR = 0.39, 95% CI: 0.23–0.65, *P* < 0.001) and TCL (HR = 0.35, 95% CI: 0.19–0.62, *P* < 0.001) patients.

**Conclusions:**

Low expression of CD5 and CD6 was associated with poor OS for TCM patients, and this may be a potential immune biomarker panel for prognostic stratification of TCM patients.

## 1. Introduction

T-cell malignancies (TCMs), including T-cell acute lymphoblastic leukemia (T-ALL) and T-cell lymphoma (TCL), are a group of heterogeneous diseases with high relapse and mortality rates. T-ALL is a malignant disease caused by clonal proliferation of precursor T lymphocytes, and it accounts for 10–15% of pediatric and 20–25% of adult ALL [1, 2]. Adult T-ALL patients have adverse clinical outcomes due to chemotherapy resistance, relapse, and no effective targeted drugs [3]. Moreover, TCL accounts for approximately 2% of non-Hodgkin's lymphoma and has a poor prognosis compared with B-cell lymphoma [4]. Gene mutations and abnormal expression are often used as prognostic biomarkers for risk stratification of cancer patients [5–7]. Recently, abnormal expressions of costimulatory and inhibitory molecules were also used as prognostic biomarkers for hematological malignancies [8, 9]. Hence, novel biomarkers derived from genetics for prognostic stratification and the development of targeted therapy in TCM are urgently needed to solve this dilemma.

Costimulatory receptor-mediated signaling has a strong impact on T-cell responses. For example, CD28, ICOS, CD40, and CD58 are potential biomarkers for prognosis and immunotherapy for hematological malignancies [10–13]. Our previous publication demonstrated that low CD58 expression is associated with poor clinical outcomes for cytogenetically normal acute myeloid leukemia (AML) patients [11]. CD5 and CD6 are class I scavenger receptors that have highly homologous extracellular regions but little conserved cytoplasmic tails [14, 15]. CD5 and CD6 are transmembrane glycoproteins that are highly similar in structure and function [16]. These proteins are expressed on the surface of the same lymphocyte populations, including mature T cells and B1 cells [17, 18]. Both are involved in the development, activation, differentiation, and survival of lymphocytes [19, 20]. Multiple studies have demonstrated that CD5 and CD6 act as costimulatory molecules for lymphocyte activation and proliferation based on monoclonal antibody experiments [20, 21]. These findings were subsequently disputed by reports of their negative modulatory effects on activation signals in CD5 and CD6 deficient mice [22–24]. Currently, there are bidirectional roles of CD5 and CD6 in cancer immunity. CD5 and CD6 have been demonstrated to affect the immune response to cancers. Furthermore, higher CD5 and CD6 expression predicts favorable outcomes for patients with nonsmall cell lung cancer (NSCLC) and plays positive roles in immune surveillance [25]. In contrast, down-regulation of CD5 expression in tumor-infiltrating lymphocytes was reported to improve the anti-tumor response in lung cancer patients [26]. Additionally, different functional variations of CD5 are related to either favorable or poor prognosis in patients with chronic lymphocytic leukemia [27]. However, the prognostic importance of CD5 and CD6 expression in TCM patients remains unclear.

In this study, peripheral blood (PB) samples from 25 *de novo* patients with T-ALL in our clinical center and transcriptome sequencing data from 131 to 162 patients with peripheral TCL (PTCL) from the GSE19069 and GSE58445 dataset of Gene Expression Omnibus (GEO) database, respectively, were obtained to investigate the prognostic value and expression levels of costimulatory molecules (CMs) in TCM.

## 2. Methods and Materials

### 2.1. T-ALL Samples

**Figure 1:**
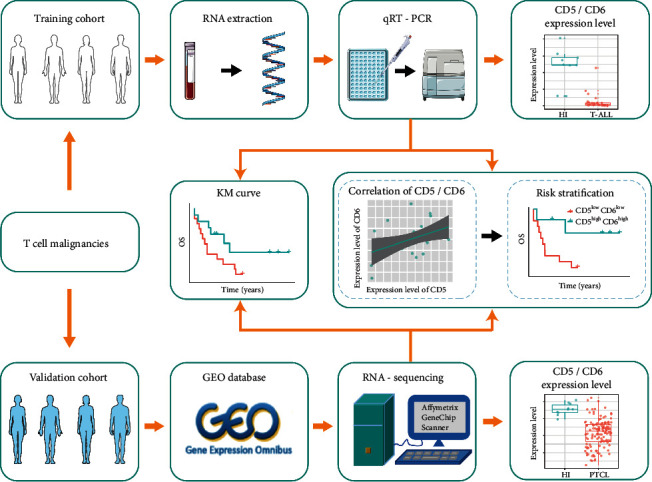
Schematics of the study. Patients with T-cell malignancies (TCMs) were divided into training (T-cell acute lymphoblastic leukemia; T-ALL) and validation (T-cell lymphoma; TCL) cohorts. Peripheral blood from T-ALL patients in the training cohort was collected for ribonucleic acid (RNA) extraction and quantitative real-time polymerase chain reaction (qRT-PCR) to detect the expression levels of CD5 and CD6. Then, the relationship between CD5 and CD6 with overall survival (OS) and co-expression of CD5/CD6 for risk stratification was analyzed. Finally, gene expression omnibus (GEO) datasets, acting as a validation cohort, were used to validate the results in the training cohort.

PB samples were collected from 25 *de novo* patients with T-ALL in our clinical center (JNU) from July 2009 to August 2016, and this was designated as a training cohort (Figure 1). The median follow-up time for surviving T-ALL patients was 7.3 years, and their clinical information is summarized in Table S1. In addition, PB samples from 9 healthy individuals (HIs) were obtained for controls. All participants provided written informed consent, and this study was conducted according to the Declaration of Helsinki principles and approved by the Ethical Committee of Jinan University.

### 2.2. Publicly Available Datasets

The GSE19069 dataset, including 10 normal T-cell samples and 131 PTCL samples, was downloaded from the GEO database (https://www.ncbi.nlm.nih.gov/geo/) to analyze the expression levels of the CMs [28]. Moreover, the GSE58445 dataset, including transcriptome data and clinical information from 162 PTCL patients, was also obtained from the GEO database [29]. Data from the GSE58445 dataset were used as the validation cohort. The clinical characteristics, including overall survival (OS) time, event, age, and gender, are listed in Table S1.

### 2.3. Quantitative Real-Time Polymerase Chain Reaction (qRT-PCR)

PB mononuclear cells (PBMCs) isolated from T-ALL patients and HI CD3+ T cells positively selected by human CD3 microbeads (Miltenyi Biotec, Bergisch. Gladbach, Germany) were extracted with TRIzol reagent (Invitrogen, Carlsbad, California, USA) according to the manufacturer's instructions [30]. Total RNA was reverse transcribed with a complementary DNA (cDNA) synthesis kit (Applied Biosystems, Foster, CA, USA). The messenger RNA (mRNA) expression levels of CD5, CD6, CD3D, CD3E, CD3G, CD247, CD4, CD8A, and CD8B were detected by a qRT-PCR kit (TIANGEN, Beijing, China) according to the manufacturer's instructions [8]. The qRT-PCR reaction procedures were as follows: preincubation, 95°C for 3 min, and amplification, 95°C for 5 sec and 60°C for 15 sec for a total of 45 cycles. mRNA expression levels were normalized to *β*-actin using the 2^−ΔΔCT^ method. The sequences of the primers are presented in Table S2.

### 2.4. Statistical Analysis

All statistical analyses were performed using Statistical Package for Social Science (SPSS) (version 26.0, Chicago, USA) software and *R* (version 4.1.3, https://www.r-project.org/). The prognostic cut-off values for quantitative variables were calculated by X-tile software (version3.6.1, Yale University, New Haven, CT, USA) [8, 31]. Kaplan–Meier curves were plotted using the *R* package “survival,” and differences between subgroups were compared by the log-rank test [5]. Univariate and multivariate Cox regression models were constructed by SPSS. Wilcoxon test (two-tailed) or Spearman method were used to evaluate the difference or correlation between two groups of quantitative data, respectively. *P* value <0.05 was regarded as statistically significant.

## 3. Results

### 3.1. Low CD5 and CD6 Expression Is Associated with Poor OS for Patients with  TCM

**Figure 2:**
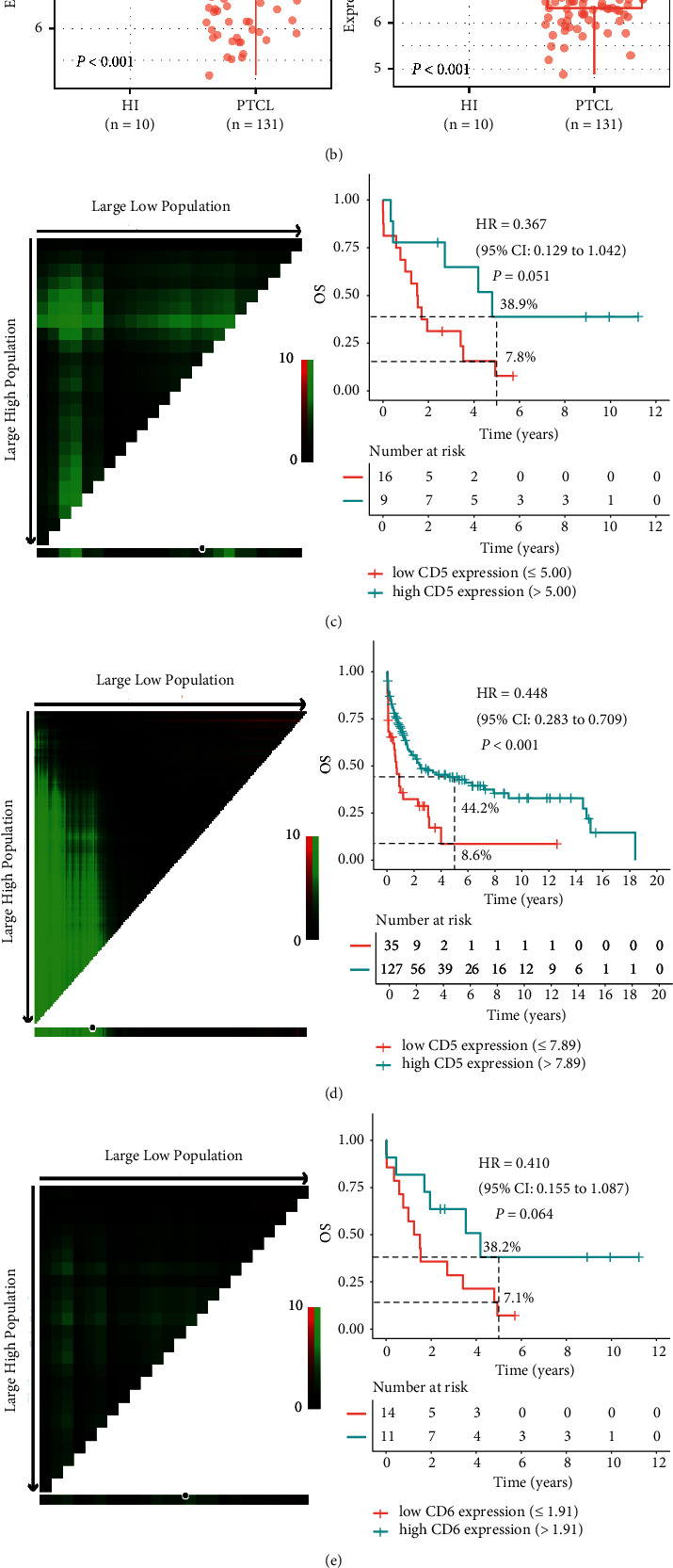
OS analysis of CD5 and CD6 in TCM patients in the training and validation cohorts. (a-b) Comparison of the CD5 and CD6 expression levels in healthy individuals (HIs) and T-ALL (a) or TCL (b) patients. (c-d) After the cut-off values were determined by X-tile software (left panel), Kaplan–Meier curves (right panel) were plotted according to subgroups of low and high CD5 expression in the training (c) and validation (d) cohorts. (e-f) Based on the cut-off values for CD6 (left panel), the TCM patients were divided into low and high CD6 expression groups, and the Kaplan–Meier curves (right panel) were plotted in the training (e) and validation (f) cohorts.

To identify prognostic CM predictors of OS in TCM patients, we first performed a Kaplan–Meier survival analysis using the GSE58445 dataset. Interestingly, a total of seven CMs including CD5, CD6, CD2, CD40, CD80, CD86, and ICOS were significantly associated with the OS for TCL patients (*P* < 0.05, Figure S1(a)). Among these CMs, CD5 and CD6 had the highest pairwise correlation; thus, CD5 and CD6 were targeted for subsequent analysis in this study (Figure S1(b)). Compared with HIs, both CD5 and CD6 were significantly down-regulated in T-ALL patients, which was confirmed in TCL patients (*P* < 0.001, Figures 2(a) and 2(b)). Importantly, T-ALL patients with low CD5 expression were associated with poor OS (hazard ratio (HR) = 0.367, 95% confidence interval (CI): 0.129–1.042; 5-year OS: 7.8% vs. 38.9%, *P* = 0.051) (Figure 2(c)). These results were also confirmed in TCL patients (HR = 0.448, 95% CI: 0.283–0.709; 5-year OS: 8.6% vs. 44.2%, *P* < 0.001) (Figure 2(d)). Moreover, T-ALL patients with low CD6 expression tended to have poor OS than those with high CD6 expression (HR = 0.410, 95% CI: 0.155–1.087, *P* = 0.064; 5-year OS: 7.1% vs. 38.2%, Figure 2(e)), and this finding was again confirmed in TCL patients (HR = 0.636, 95% CI: 0.419–0.996, *P* = 0.032; 5-year OS:30.3% vs. 41.8%, Figure 2(f)). Due to the small T-ALL sample size, subgroup analysis was performed for only the TCL patients. Notably, lower CD5 expression was significantly associated with poor OS in TCL patients greater than 60 years old (HR = 0.375, 95% CI: 0.197–0.714, *P* = 0.002; 5-year OS: 0 vs. 38.7%). The same result was also found for CD6 (HR = 0.551, 95% CI: 0.303–1.003, *P* = 0.048; 5-year OS: 22.8% vs. 35.7%) (Figures S2(a)–S2(d)).

**Figure 3:**
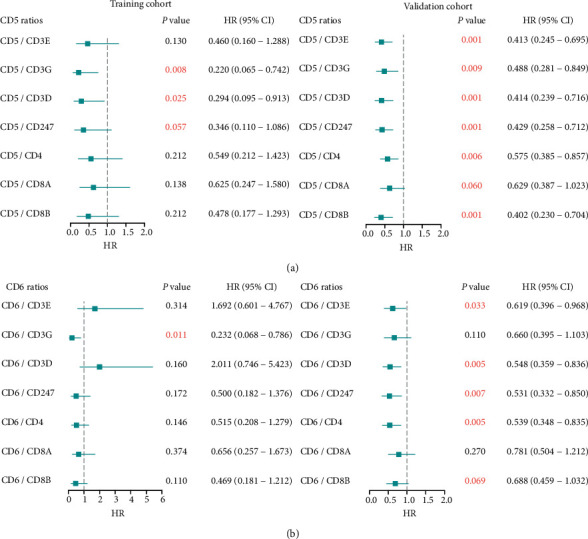
OS analysis of the CD5 and CD6 ratios in the training and validation cohorts. (a-b) OS analysis was performed with the dichotomized relative expression ratios of CD5/CD3E, CD5/CD3G, CD5/CD3D, CD5/CD247, CD5/CD4, CD5/CD8A, CD5/CD8B (a) and the CD6/CD3E, CD6/CD3G, CD6/CD3D, CD6/CD247, CD6/CD4, CD6/CD8A, CD6/CD8B ratios (b) in the training (left panel) and validation (right panel) cohorts.

CD5 and CD6 are constitutively expressed lymphocyte receptors whose expression can be regulated during lymphocyte development and activation events. Therefore, correlations with the up-regulated CD3, CD4, and CD8 expression levels in T cells were evaluated, which would relatively exclude the effects of T-cell counts on CD5 and CD6 expression. The gene expression of CD5 and CD6 was normalized to that of CD3E, CD3G, CD3D, CD247, CD4, CD8A, and CD8B. Interestingly, higher CD5/CD3G (HR = 0.220, *P*=0.008), CD5/CD3D (HR = 0.294, *P*=0.025), and CD5/CD247 (HR = 0.346, *P*=0.057) expression was associated with improved OS in T-ALL patients (Figure 3(a), left panel). These results were confirmed in TCL patients (CD5/CD3G: HR = 0.488, *P*=0.009; CD5/CD3D: HR = 0.414, *P*=0.001; CD5/CD247: HR = 0.429, *P*=0.001) (Figure 3(a), right panel). Moreover, higher CD5/CD3E, CD5/CD4, CD5/CD8A, and CD5/CD8B expression was associated with favorable OS for TCL patients (HR < 1, *P* ≤ 0.06) (Figure 3(a), right panel). Additionally, T-ALL patients with higher CD6/CD3G expression had a favorable OS, while TCL patients with higher CD6/CD3E, CD6/CD3D, CD6/CD247, CD6/CD8, and CD6/CD8B (HR < 1, *P* < 0.07) had favorable outcomes (Figure 3(b)). On the contrary, CD6/CD8A had no significant association with OS (Figure 3(b)).

### 3.2. Co-Expression of CD5/CD6 for Prognostic Stratification in TCM Patients

**Figure 4:**
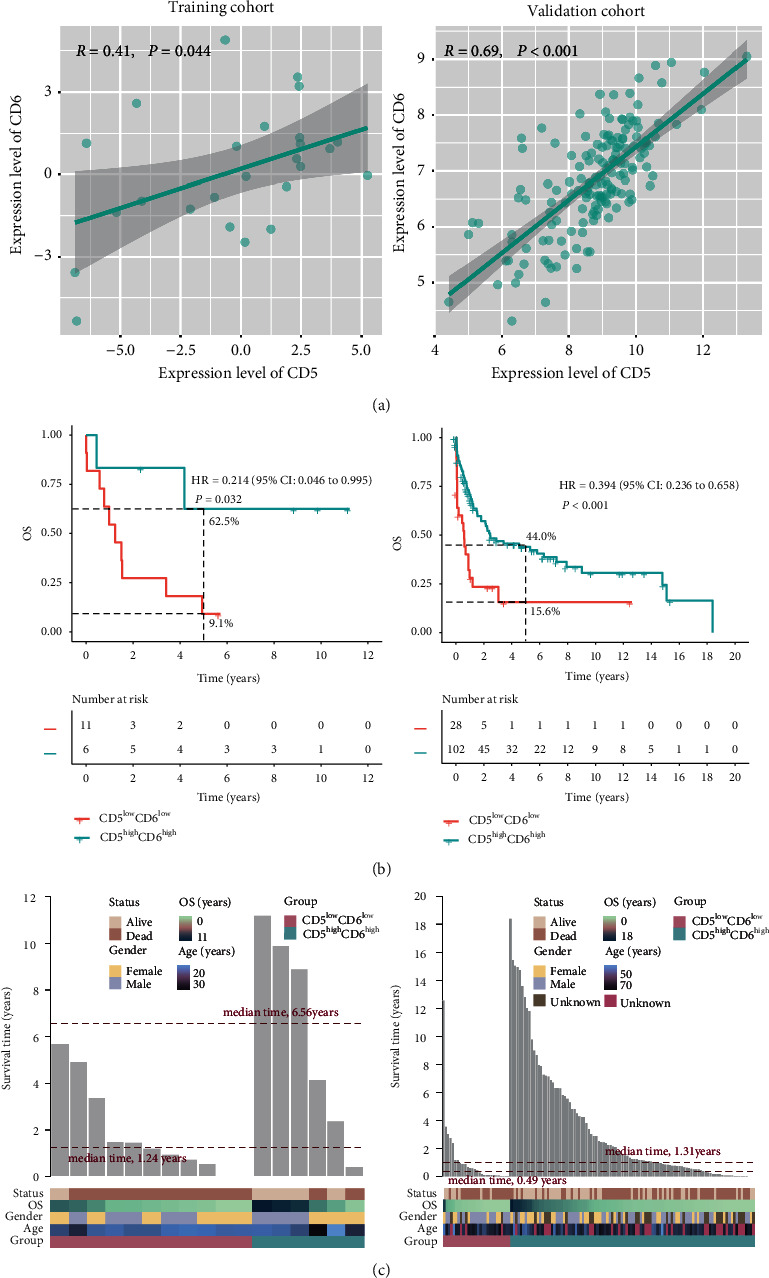
Co-expression of CD5 and CD6 for prognostic stratification in TCM patients. (a) Correlation between the CD5 and CD6 expression levels in the training (left panel) and validation (right panel) cohorts. (b) Kaplan–Meier curves for patients who were CD5^high^CD6^high^ or CD5^low^CD6^low^ in the training (left panel) and validation (right panel) cohorts. (c) Distribution of the OS time for patients who were CD5^high^CD6^high^ or CD5^low^CD6^low^ in the training (left panel) and validation (right panel) cohorts.

Because CD5 and CD6 are co-receptors on the surface of lymphocytes, their correlation was investigated. We demonstrated a strong positive relationship between CD5 and CD6 in TCL patients, and this was also found in T-ALL patients (*R* = 0.41, *P* = 0.044) (Figure 4(a)). Combinations of genes may be better than a single gene in predicting prognoses and performing risk stratification for cancer patients. Interestingly, T-ALL patients who were CD5^low^and CD6^low^ had poor OS (HR = 0.214, 95% CI: 0.046–0.995, *P* = 0.032; 5-year OS: 9.1% vs. 62.5%) and a shorter median OS (1.24 vs. 6.56 years) than those who were CD5^high^and CD6^high^ (Figures 4(b)-4(c), left panel). Similar findings were shown in TCL patients (HR = 0.394, 95% CI: 0.236–0.658, *P* < 0.001; 5-year OS: 15.6% vs. 44.0%; median OS: 0.49 vs. 1.31 years) (Figures 4(b)-4(c), right panel). In addition, when sex, age, and CD5/CD6 ratio were included in univariate and multivariate COX regression models for survival analysis, the results indicated that CD5^high^CD6^high^ was an independent prognostic predictor of OS in T-ALL patients (HR = 0.39, 95% CI: 0.23–0.65, *P* < 0.001). This finding was confirmed in TCL patients (HR = 0.35, 95%CI: 0.19–0.62, *P* < 0.001) (Table 1).

## 4. Discussion

Risk stratification based on the International Prognostic Index (IPI) (including age, stage, performance status, serum lactate dehydrogenase level, and extranodal involvement) has made great progress in predicting the prognosis of patients with TCL, who can be divided into four groups: low, low-intermediate, high-intermediate, and high-risk [32]. This precise risk stratification can provide important references for the management of TCL patients and clinical decision-making, thereby improving patient outcomes [32, 33]. However, IPI-based risk stratification cannot accurately predict a prognosis for all TCL patients [33, 34]. The reasons for this heterogeneity may be due to clinical characteristics, morphology, genetics, and immunophenotype. Notably, gene alterations play an important role in constructing risk stratification for hematological malignancies, particularly acute myeloid leukemia, but there is a lack of information on the genetic alterations that complement risk stratification to more accurately predict clinical outcomes for TCL patients [32, 35, 36]. In addition, although multiple studies have been actively exploring the role of genetic alterations combined with minimal residual disease (MRD) and clinical information in risk stratification for T-ALL patients, high heterogeneity makes it difficult to accurately stratify all patients [37]. Therefore, further exploration of novel biomarkers to improve risk stratification for TCM patients is needed.

Previous studies have reported that some CMs can be used as prognostic biomarkers for hematological malignancies. AML patients with B7-2 positivity shared a poorer prognosis compared to AML patients who were B7-2 negative [38]. In this study, eleven CMs were analyzed, and seven were associated with the prognosis of TCM patients. However, different hematological malignancies may have different immune receptor abnormalities, and the combination of two related immune receptors has greater advantages compared with a single molecule in predicting the prognosis of patients [5, 6, 8, 11]. Interestingly, CD5 had the strongest correlation with CD6, and low expression of CD5 and CD6 was significantly associated with adverse outcomes in TCM patients. These findings were consistent with the results that low CD5 and CD6 expression predicts poor prognosis in patients with NSCLC or melanoma [25]. TCL patients older than 60 years of age have adverse clinical outcomes and higher risk stratification compared with those younger than 60 [32]. Thus, more precise stratification is required for TCL patients older than 60 years for rational decision-making. Interestingly, our study suggests that low expression of CD5 and CD6 could predict poor OS in TCL patients older than 60 years. However, due to the small T-ALL sample size, subgroup analysis could not be performed for validation.

CD5 and CD6 are transmembrane glycoproteins expressed on the surface of T cells that act as costimulatory molecules in the TCR signaling pathway [39, 40]. TCMs are transformed by the malignant proliferation of T cells and an increase in T-cell counts. To this end, we normalized the CD5 and CD6 levels to that of the mRNA expression of CD3, CD4, and CD8 [25]. Interestingly, even after normalization, the expression of CD5 and CD6 predicted the prognosis of TCM patients but was not affected by the T-cell counts. Altogether, CD5 and CD6 might be immune biomarkers for the prognostic stratification of TCM patients. We attempted to validate our findings with additional T-ALL and TCL publicly available datasets, but we could not obtain the complete prognostic information and transcriptome data for necessary analysis; thus, more T-ALL or TCL samples are needed to further validate our results in the future.

In conclusion, we observed that lower expression of CD5 and CD6 was associated with poor OS for patients with TCM, and co-expression of CD5 and CD6 was an independent prognostic predictor of OS in TCM patients. These findings provided deep insight that CD5 and CD6 might be immune biomarkers for prognostic stratification and the development of targeted therapies for TCM patients.

## Figures and Tables

**Table 1:** Univariate and multivariate cox regression analysis in TCM patients.

Variables	Univariate cox regression				Multivariate cox regression			
	Training cohort		Validation cohort		Training cohort		Validation cohort	
	HR (95% CI)	*P* value	HR (95% CI)	*P* value	HR (95% CI)	*P* value	HR (95% CI)	*P* value
Sex (ref: female)^*∗*^								
Male	0.68 (0.27, 1.71)	0.412	1.60 (0.99, 2.61)	0.057	0.96 (0.36, 2.56)	0.934	1.62 (0.98, 2.68)	0.060
Age, years^*∗*^								
	0.99 (0.96, 1.02)	0.564	1.02 (1.01, 1.04)	0.001	0.98 (0.95, 1.02)	0.395	1.03 (1.01, 1.04)	0.001
CD5/CD6 (ref: CD5lo/CD6 lo) ^*∗*^								
CD5hi or CD6 hi	0.86 (0.32, 2.32)	0.765	0.44 (0.24, 0.83)	0.011	0.95 (0.34, 2.68)	0.922	0.41 (0.21, 0.80)	0.010
CD5hi/CD6 hi	0.19 (0.04, 0.86)	0.031	0.39 (0.23, 0.65)	< 0.001	0.18 (0.04, 0.88)	0.034	0.35 (0.19, 0.62)	< 0.001

^*∗*^ Cox regression analysis of data with complete age and gender information. CI: confidence interval; HR: hazard ratio.

## Data Availability

The GSE58445 and GSE19069 datasets were downloaded from the Gene Expression Omnibus (GEO) dataset (https://www.ncbi.nlm.nih.gov/geo/). The datasets used and/or analyzed during the current study are available from the corresponding author on reasonable request.

## Supplementary Materials

Supplementary Materials Table S1: clinical characteristics of patients with TCM. Table S2: primers for qRT-PCR. Figure S1: prognostic (A) and correlation (B) analysis of costimulatory molecules in TCL patients. Figure S2: A–D: subgroup analysis of CD5 (left panel) and CD6 (right panel) in TCM patients younger than 60 years (A) and greater than 60 years (B), as well as female (C) and male (D) patients in the training cohort.
