# Supplementary material for: Low Expression of CD5 and CD6 Is Associated with Poor Overall Survival for Patients with T-Cell Malignancies
Source: J Oncol. 2022 Aug 9;2022:2787426. doi: 10.1155/2022/2787426 (PMC9381250; doi:10.1155/2022/2787426)
Supplement: Supplementary Materials — Table S1: clinical characteristics of patients with TCM. Table S2: primers for qRT-PCR. Figure S1: prognostic (A) and correlation (B) analysis of costimulatory molecules in TCL patients. Figure S2: A–D: subgroup analysis of CD5 (left panel) and CD6 (right panel) in TCM patients younger than 60 years (A) and greater than 60 years (B), as well as female (C) and male (D) patients in the training cohort. [file 2787426.f1.docx]

| **Table S1.** Clinical characteristics of patients with TCM. | | |  |
| --- | --- | --- | --- |
| Variables | JNU dataset | GSE58445 | GSE19069 |
| Number | 25 | 162 | 131 |
| Subtype | T - ALL | PTCL | PTCL |
| Gender (%) * |  |  |  |
| Female | 13 (52.0) | 49 (30.2) | - |
| Male | 12 (48.0) | 77 (47.5) | - |
| Unknown | 0 (0) | 36 (22.2) | - |
| Age, years, mean ± SD  Median OS time,  range, years | 30.0 ± 16.7  1.95  (0.006 - 11.222) | 58.7 ± 19.0  8.99  (0.003 - 18.400) | -  -  - |
| Event (%) |  |  | - |
| Dead | 19 (76.0) | 98 (60.5) | - |
| Alive | 6 (24.0) | 64 (39.5) | - |

* Due to rounding, not all percentages total 100%.

SD: Standard deviation.

OS: Overall survival.

**Table S2.** Primers for qRT-PCR.

| **Primer** | **Sequence 5’- 3’** |
| --- | --- |
| CD5 (F) | CGAGAGCCAGACCTGTTTGT |
| CD5 (R) | CCCCCGAGTCTCTAGTTGGA |
| CD6 (F) | ACTACTGCGGCCACAAAGAG |
| CD6 (R) | GAAGTGTACCTCCACCTGC |
| CD4 (F) | CTTGGATCACCTTTGACCTGA |
| CD4 (R) | CTCATCACCACCAGGTTCACT |
| CD8A (F) | GAGACAGTGGAGCTGAAGTGC |
| CD8A (R) | AGGGCCGAGCAGAAATAGTAG |
| CD8B (F) | TGGGATTCCGCAAAAGGGAC |
| CD8B (R) | CTTCACGCTTGTGAGATTGAGAA |
| CD3D (F) | TCATTGCCACTCTGCTCC |
| CD3D (R) | GTTCACTTGTTCCGAGCC |
| CD247 (F) | GCCAGAACCAGCTCTATAAC |
| CD247 (R) | TAGGCCTCCGCCATCTTATC |
| CD3E (F) | TCCCAACCCAGACTATGAGC |
| CD3E (R) | CAAGACTAGCCCAGGAAACAG |
| CD3G (F) | GGGATGTATCAGTGTAAAGG |
| CD3G (R) | CAGCAATGAAGTAGACCC |


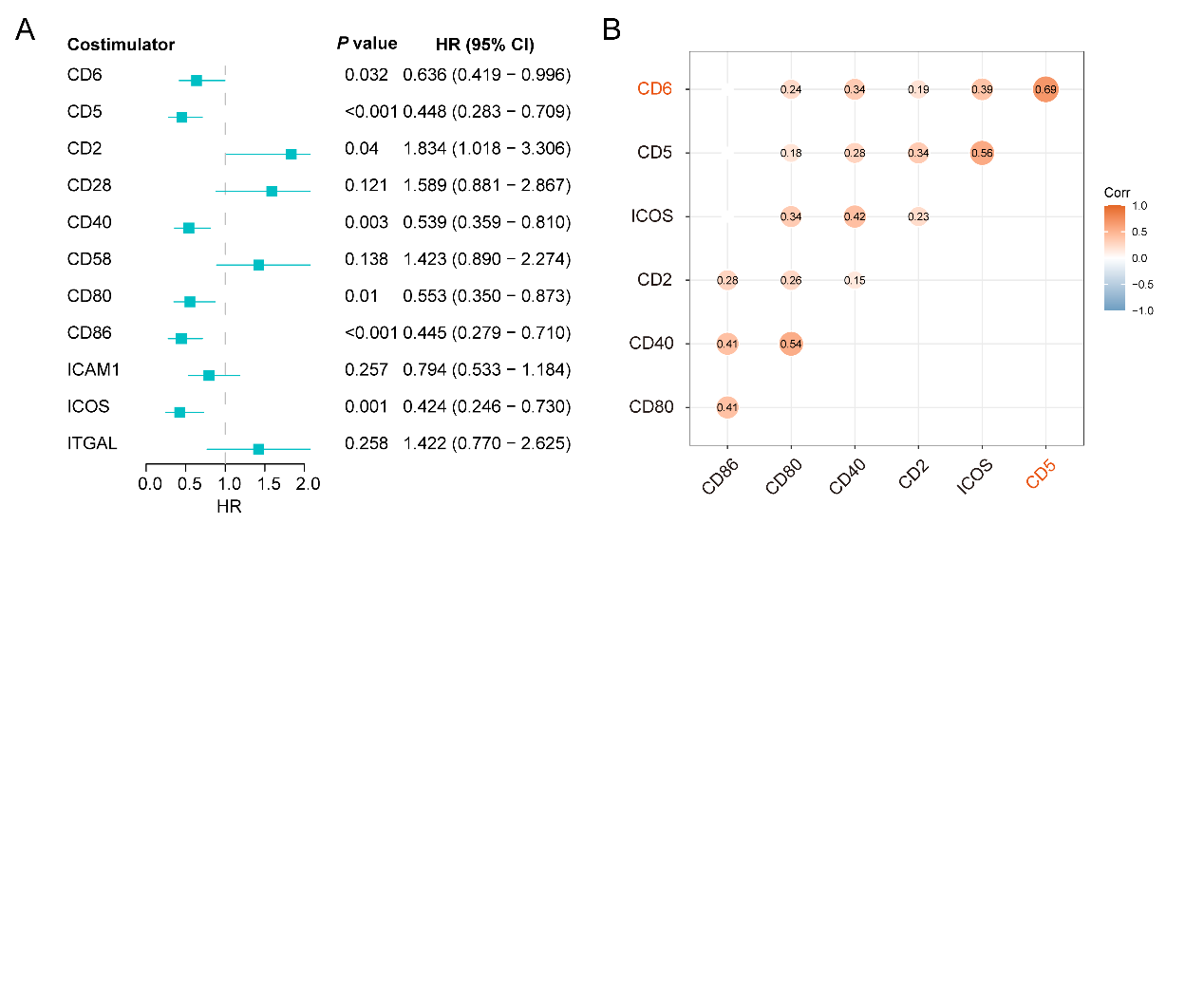


**Figure S1.** Prognostic (A) and correlation (B) analysis of costimulatory molecules in TCL patients.


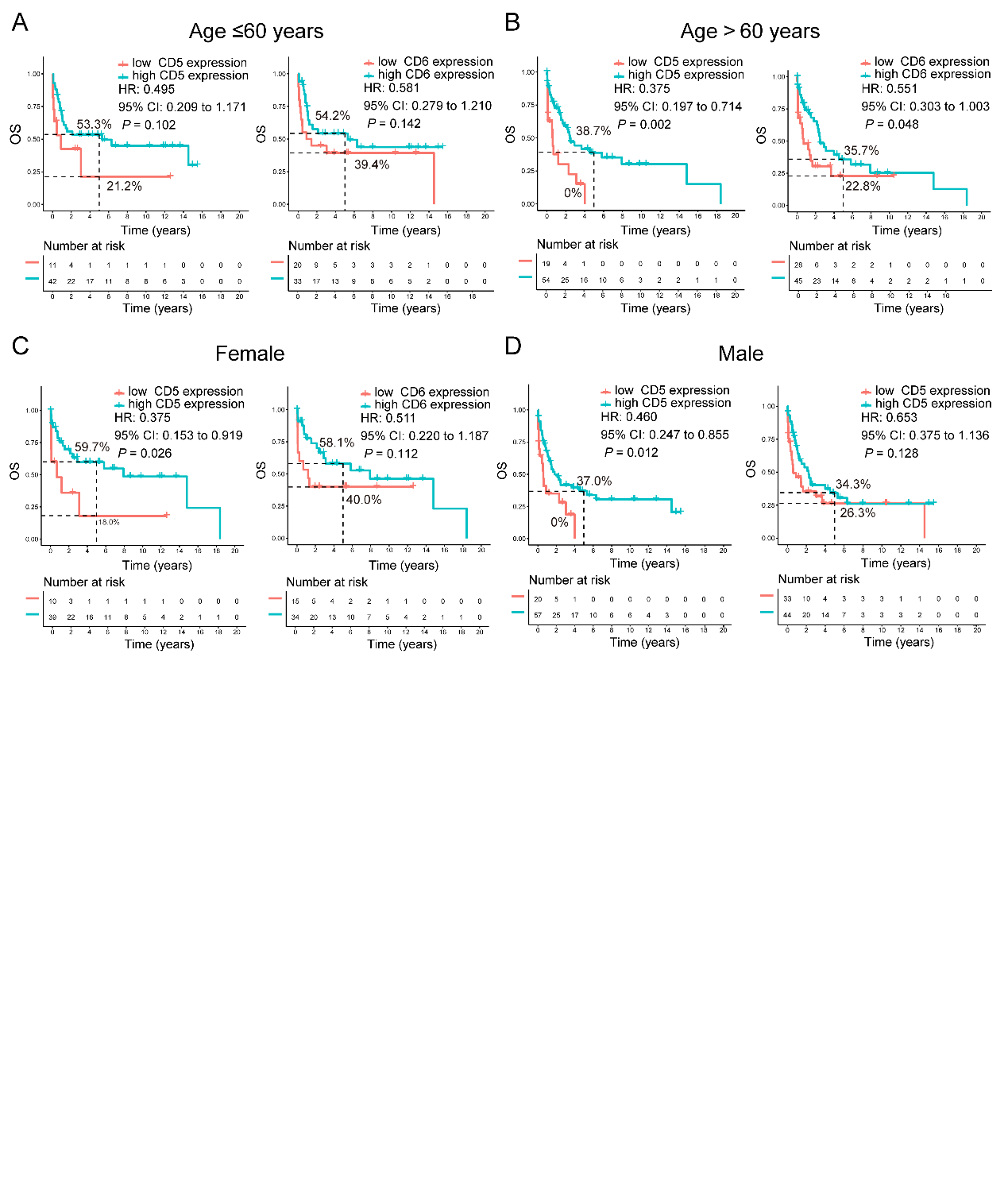


**Figure S2.** A-D: Subgroup analysis of CD5 (left panel) and CD6 (right panel) in TCM patients younger than 60 years (A) and greater than 60 years (B), as well as female (C) and male (D) patients in the training cohort.
